# A Novel Blocking ELISA for Detection of Antibodies against Hepatitis E Virus in Domestic Pigs

**DOI:** 10.1371/journal.pone.0152639

**Published:** 2016-03-29

**Authors:** Yiyang Chen, Qin Zhao, Baoyuan Liu, Lizhen Wang, Yani Sun, Huixia Li, Xinjie Wang, Shahid Faraz Syed, Gaiping Zhang, En-Min Zhou

**Affiliations:** 1 Department of Preventive Veterinary Medicine, College of Veterinary Medicine, Northwest A&F University, Yangling, Shaanxi, China; 2 Scientific Observing and Experimental Station of Veterinary Pharmacology and Veterinary Biotechnology, Ministry of Agriculture, Yangling, Shaanxi, China; 3 College of Animal Science and Veterinary Medicine, Henan Agricultural University, Zhengzhou, Henan, China; CEA, FRANCE

## Abstract

Hepatitis E virus (HEV) infects both humans and animals, with an overall human mortality rate generally less than 1%, but as high as 20% among pregnant women. HEV strains fall into 4 major genotypes. Zoonotic genotypes 3 and 4 associate with sporadic human and animal HEV cases in many industrialized countries. To date, collective evidence implicates pigs as the main HEV reservoir, justifying the importance of monitoring HEV infection rates in pig herds to prevent human illness. Due to the lack of a robust *in vitro* cell culture system for viral propagation, no “gold standard” assay has yet been developed to detect HEV infection in domestic pigs. 1E4, a monoclonal antibody (mAb) specific for the C-terminal 268 amino acids of HEV genotype 4 ORF2 capsid protein (sORF2-C), was generated and conjugated to horseradish peroxidase (HRP) for use in a blocking ELISA (bELISA). Optimal sORF2-C coating antigen concentration (8 μg/ml), HRP-1E4 dilution (1:1000), and test pig serum dilution (1:20) were determined using a checkerboard titration test. A cut-off value of 16.9% was chosen to differentiate between positive vs. negative sera after mean percent inhibition (PI) testing of 230 negative pig sera. Compared with the indirect ELISA (iELISA), western blot, and a commercial ELISA kit for detecting anti-HEV antibodies in human sera, the bELISA showed no statistical differences and statistically high coincidence of 93.23%, 92%, and 95% with the other tests, respectively. A blocking ELISA (bELISA) for detecting anti-HEV antibodies in pig serum samples was developed with high sensitivity and high specificity comparable to that of the indirect ELISA. The bELISA results exhibited high agreement with iELISA, western blot, and a commercial ELISA kit designed to detect human anti-HEV antibodies. Therefore, bELISA should serve as an ideal method for large-scale serological investigation of anti-HEV antibodies in domestic pigs.

## Introduction

Hepatitis E virus (HEV) can cause self-limiting hepatitis in humans that is a serious public health problem in many developing countries, as well as some developed countries [1]. Generally, 1–4% of HEV infections can lead to fulminant hepatitis in humans, but in some endemic regions infection rates can be as high as 20% in pregnant women [2]. Moreover, many case reports have described chronic cases of hepatitis E in immunosuppressed individuals [3]. HEV, which belongs to the *Hepeviridae* family, is a non-enveloped positive-sense, single-stranded RNA virus [4, 5]. The viral genome is about 7.2 kb in size and contains three open reading frames (ORFs), ORF1, ORF2, and ORF3 [6]. ORF2 encodes a capsid protein of 660 amino acids and contains the primary epitopes of the viral particles and is used universally as the antigen for detection of antibodies against HEV [4, 7].

In addition to humans, HEV strains have also been isolated from wild and domestic pig [8], wild boar [9], deer [9], chicken [10, 11], rat [12], ferret [13], rabbit [14], mongoose [9], camel [15], and bat [16]. The HEV isolates from human, pig, wild boar, deer, mongoose, rabbit, and camel have been taxonomically classified into the *Orthohepevirus* A and include 4 major genotypes (HEV-1 to HEV-4) [17]. Genotypes 3 and 4 (HEV-3 and HEV-4) have been isolated from humans and many animal species and are associated with sporadic cases of hepatitis E in many industrialized countries. To date, accumulating evidence indicates that hepatitis E is a zoonotic disease and that pigs and wild boars are the main HEV reservoirs.

Currently, the global burden of HEV human infection is thought to largely comprise sporadic hepatitis E cases, with a higher infection rate observed for workers in close occupational proximity to swine, swine manure, or sewage [18–20]. Therefore, to prevent human HEV infection, it is very important to measure HEV infection rates in pig herds [5]. Moreover, development of an FDA-approved diagnostic assay should be a priority. Since pigs naturally infected with swine HEV are asymptomatic [1], the diagnosis of swine HEV infection must rely on RT-PCR and serology [21–24]. For serological diagnosis in pigs, anti-HEV antibodies are universally detected using two methodologies: commercial kits optimized for detection of human anti-HEV antibodies that have been adapted to use for swine and in-house indirect enzyme-linked immunosorbent assays (iELISAs) using genotype 3 or 4 HEV ORF2 proteins as coating antigens [24–26]. Unfortunately, these iELISAs have not yet been validated, due to the absence of appropriate “gold standard” for comparison [1] and studies have shown that iELISAs often provide discordant results and non-specific background signals [27]. In contrast, monoclonal antibody-based blocking ELISA (bELISA) could decrease non-specific binding artifacts and improve the specificity of detection of antibodies in serum samples.

In this study, a bELISA for detection of porcine anti-HEV serum antibodies was developed using the truncated ORF2 protein of genotype 4 swine HEV as coating antigen. Detection of serum antibodies specific for ORF2 was performed by observing their inhibition of binding of HRP-conjugated monoclonal antibody to the coating antigen. As compared with the iELISA, which also used a truncated ORF2 coating antigen, the bELISA developed in this work showed the same sensitivity and higher specificity. In addition, statistically the bELISA results exhibited very high coincidence with iELISA, western blot, and a commercial ELISA kit for detecting human anti-HEV antibodies. On the basis of these findings, the bELISA should serve as a useful method for large-scale serological investigation of anti-HEV antibodies in pigs.

## Materials and Methods

### Serum samples

To validate the bELISA developed in this study, a total of 45 serum samples were collected from 5 pigs at 0, 7, 14, 21, 28, 35, 42, 49, and 56 days post inoculation (dpi) with CHN-SD-sHEV (genotype 4, GenBank accession number KF176351). The weaning piglets (Landrace × Yorkshire) at 2 month of age (initial body weight: 10.7 [S.D. 1.74] kg) were used in this study. One week before challenged, pigs were moved into the barn equip heating lamps and solid floor with a deep litter bedding of straw and wood shavings in which they remained until the end of the study. Space allowance in housing conditions was about 1.0–1.2 m^2^ per pig. Pure water and dry pelleted feed was available ad libitum. The room temperature was about 20 to 21°C, and the ambient temperature was recorded every day. A 12-h light/dark cycle was given for pigs get enough sleep. As environmental enrichment, pigs received some soft nontoxic plastic balls linked with metal chain as chewing material. After these pigs passed the adjustment period, they were inoculated via ear vein with 200 μl of CHN-SD-sHEV infectious stock containing 10^4^ genomic equivalents (GE)/ml, except for virus injection, other details were similar to serum sampling described as followed. No pig died or became severely ill prior to the experimental endpoint. In addition, a total of 230 seronegative samples and 56 seropositive serum samples collected from clinically infected pigs were tested for anti-HEV antibodies using a commercial ELISA kit optimized for detection of human anti-HEV antibodies (Wantai Biological Pharmacy Co., Beijing, China).

For prevalence studies, 2,542 pig serum samples were collected from 8 herds in Shaanxi Province, China. Out of which, the 5 herds of Shixun (n = 871), Lijia (n = 415), Bigong (n = 350), Zhengda (n = 269) and Paisidong (n = 47) were located in Xianyang (34°36′N and 108°73′E), the two herds of Benyuan (n = 445) and Yanchuan (n = 25) were located in Yan’an (34°6′N and 109°48′E) and one herd of Jinfeng (n = 120) was located in Baoji (34°38′N and 108°15′E). The serum samples mentioned above were collected during 2011–2012 years period. All owners gave their permission to conduct the study on these sites. In addition, 94 clinical positive sera raised against other swine viruses, including porcine reproductive and respiratory syndrome virus (PRRSV), classical swine fever virus (CSFV), and porcine circovirus (PCV), were used to evaluate the specificity of the developed bELISA. These sera were separately confirmed with their respective commercial ELISA kits (IDEXX Laboratories, USA).

All above serum samples were collected via pigs ear vein and the pigs were carried out for alleviate suffering minimized. Firstly, a rubber tourniquet was placed at the base of the ear and the ear skin was scrubbed with alcohol (ethanol or xylol) to cause congestion of the ear vein. An “18-gauge” needle has been fixed onto the serum tube (A380329, Tian’ai Company, Shandong province of China) and then inserted percutaneously into the ear vein to provide access for serial blood sampling. The serum tube may be filled within a few seconds.

The animal experiments including the above pigs and the below mice were approved by the Animal Care and Use Committee of Northwest Agricultural & Forestry University (NWSUAF, Permit Number: AE189056) with adherence to NWSUAF guidelines during handling of all experimental animals.

### Prokaryotic expression and purification of recombinant swine HEV ORF2 protein

The truncated capsid protein (sORF2-C) of genotype 4 swine HEV was expressed in a bacterial system and purified as previously described [28]. Briefly, the recombinant plasmid containing the target gene encoding the C-terminal 268 amino acids of the capsid protein was transformed into *Escherichia coli BL21* (DE3). Next, expression of recombinant protein was induced at 37°C for 6 h with 1.0 mM isopropyl-β-D-thiogalactopyranoside (IPTG). The recombinant protein was dissolved in 8 M urea and purified using a high-affinity nickel-nitrilotriacetic-acid (Ni-NTA) resin column according to the manufacturer’s instructions (Jinsite Biotechnology Corp., Nanjing, China). The purified protein was analyzed by SDS-PAGE and its concentration determined using a Bradford Protein Assay Kit (Vigorous Biotechnology Co., Beijing, China) based on the manufacturer’s instructions. The purified protein was reconstituted in renaturation buffer (1 mM EDTA, pH 7.2 PBS containing 6, 4, 2, or 0 mM urea). The renatured sORF2-C was used as the immunizing antigen for production of mAbs and also as the coating antigen for the iELISA and bELISA developed in this work.

### Production, purification, and conjugation of monoclonal antibodies with horseradish peroxidase

Six 6-week-old female BALB/c mice obtained from experimental animal center of Xi’an Jiaotong University were immunized intraperitoneally with 100 μg/mouse of sORF2-C protein emulsified in complete Freund’s adjuvant (Sigma–Aldrich), followed by two more injections at two and four weeks post immunization using the same protein and dose but emulsified in incomplete Freund’s adjuvant. One month after the third injection, the mice were given a final booster injection by tail vein. The mice received the rodent AM-II diet and they were housed at the Institute Animal Core Facility in a temperature- and humidity-controlled room with a 12-h light/dark cycle and given free access to food and water continuously available. The mice body condition and health were monitored twice a day, including weigh and feces examination. In the environmental condition, no mice died and became severely ill prior to the experimental endpoint. Five days later, mice were first anesthetized with a dose of Ketamine and Acepromazine (100mg/Kg K+ 5mg/Kg A) via injecting intraperitoneally. Mice were sacrificed by cervical dislocation.

The spleen cells from the mice were fused with SP2/0 murine myeloma cells according to the standard polyethylene glycol-mediated fusion method. Hybridomas secreting antibodies against sORF2-C protein were identified using iELISA and were subsequently subcloned twice to establish stable clones. The mAbs in the tissue culture medium were purified using a goat anti-mouse IgG affinity column according to the manufacturer’s instructions (Jinsite Company, Nanjing, China). The purified mAbs were analyzed by SDS-PAGE and their concentrations were estimated by absorbance at 280 nm using a spectrophotometer (UV-2450, SHIMADZU Corporation) at an absorption coefficient of OD_280_/(1.35 mg/ml). After concentration determination, the purified mAbs were conjugated to horseradish peroxidase (HRP) using a kit according to the manufacturer’s instructions (Roche Diagnostics, Basel, Switzerland). In a final step, the mixture was dialyzed against 0.01 M PBS (pH 7.2). HRP-mAbs were adjusted to an OD_450nm_ of ~1. The titers of HRP-mAbs were measured using direct ELISA as described previously [29] and HRP-mAbs were added to assays in the range of dilutions of 10^−1^ to 10^−4^.

### Spatial relationships of epitopes recognized by mAbs

To determine the spatial relationships of epitopes recognized by the three mAbs in this study, a competitive ELISA was performed according to the procedure described previously [30, 31] with the following modifications. The purified sORF2-C protein was used as the coating antigen and the residual binding of each mAb to the solid phase-bound sORF2-C protein was detected using HRP-mAbs. Maximal binding without inhibition was found when the HRP-mAbs were added without competitors.

### Selection of one HRP-mAb for development of the blocking ELISA

For bELISA development, the three HRP-mAb candidates were tested to determine which mAb could best compete with known seropositive pig serum samples for antigen binding. Specifically, the 45 serum samples collected 0 to 56 dpi from the 5 pigs challenged with CHN-SD-sHEV (genotype 4) were used to block binding of the three HRP-mAbs to sORF2-C protein bound to a solid phase. The HRP-mAb exhibiting the highest ability to overcome serum antibody competition for antigen binding, as measured by the highest ratio of the percent inhibition (PI) of binding of seropositive sera (the 7 to 56 dpi serum samples) to seronegative sera (0 dpi control serum samples), was selected for bELISA development. The bELISA procedures are described below.

### Development of the blocking ELISA

Optimal concentrations of the coating antigen and dilutions of the selected HRP-mAb were determined through direct ELISA using a checkerboard titration test. The sORF2 protein was used at concentrations 2 μg/mL, 4 μg/mL, and 8 μg/mL and HRP-mAb was tested using dilutions at 1:100, 1:1000, and 1:10000. The optimal antigen concentration and HRP-mAb dilution used for the bELISA, each with an OD_450nm_ value of ~1.0, were determined using the direct ELISA.

To determine the optimal dilutions of test serum samples, one positive and one negative pig serum sample were each diluted 1:10, 1:20, and 1:40 for testing by bELISA. The dilutions ultimately chosen for the test serum samples were determined from the dilutions of the positive and negative pig serum samples that produced the highest PI based on the formula described below. After optimizing the above conditions, MaxiSorp microtiter plates (Nunc A/S, Roskilde, Denmark) were coated using an optimal concentration (8 μg/mL) of sORF2-C protein (100 μL/well) in 0.01 M PBS (pH 7.2) and incubated overnight at 4°C. Antigen-coated plates were washed three times with PBST (PBS containing 0.5% (v/v) Tween-20) and nonspecific binding sites were blocked with 200 μL of blocking buffer (2.5% (w/v) non-fat dry milk in PBST) for 1 h at room temperature (RT). After 3 washes with PBST, 100 μL of test samples, either positive or negative serum samples diluted in blocking buffer, were added separately to each well in duplicate. Next, the plates were incubated for 1 h at RT followed by three washes and addition of 100 μL/well of mAb IE4-HRP at the optimized dilution (1:1000), with incubation at RT for an additional 1 h. Following a final three washes, 100 μL/well of 3,3',5,5'-Tetramethylbenzidine (TMB) substrate, made from mixing of two solutions, A and B (A: 205 mM potassium citrate (pH 4.0); B: 41 mM TMB) in a ratio of A: B (v/v) of 39:1 was added to each well and the plates were incubated in the dark for 15 min at RT. As a final step, 3 M H_2_SO_4_ (50 μL/well) was used to stop the colorimetric reaction and the OD_450nm_ values were read using an automated ELISA plate reader (Bio-Rad, USA).

### Validation of the blocking ELISA

The PI values for the test serum samples were determined using the following formula: PI (%) = (1 − (OD_450nm_ of test serum samples/OD_450nm_ value of negative control serum samples)) × 100%. The 230 HEV antibody-negative pig serum samples, confirmed by a commercial ELISA kit for detecting human anti-HEV antibodies, were used to determine the cut-off value between positive and negative serum samples. The cut-off value for the bELISA was chosen based on the mean PI value determined from the 230 negative serum samples plus 3 standard deviations (SD), which would ensure that 99% confidence for the negative sera samples fell within this range.

The sensitivity of bELISA was evaluated by testing 45 serum samples from across the overall dpi range of the 5 challenged pigs, as well as the 56 clinical positive serum samples that had been confirmed using the commercial ELISA kit. The specificity of the cross-blocking assay comprising the bELISA was performed by comparing results for HEV seropositive samples results for clinical positive antisera against other swine viruses, including PRRSV, CSFV, and PCV.

The reproducibility of the bELISA was evaluated by testing six samples selected from the 56 clinical positive serum samples tested by the commercial ELISA kit. The OD_450nm_ values of the 56 samples were sorted from low to high (0.3–0.4, 0.4–0.5, 0.5–0.6, 0.6–0.7, 0.7–0.8 and >0.8) and divided into six groups. One sample from each group was used as the positive serum sample to perform the intra-assay and inter-assay variabilities. The coefficient of variation (CV) was used to evaluate the inter-assay variation (between plates) and the intra-assay variation (within a plate). Each sample was tested using three different plates tested on different occasions to determine the inter-assay CV and three replicates within each plate were used to calculate the intra-assay CV.

### Comparisons of blocking ELISA with indirect ELISA, western blot, and commercial ELISA kit

To test the coincidence of the bELISA with the iELISA, 2,542 clinical pig serum samples were tested using both methods. The procedures and cut-off value of the iELISA were described in Wang et al. [28]. Several serum samples exhibiting positive but inconsistent results between the iELISA and bELISA were further tested using western blot and the commercial ELISA kit. The western blot procedure was based on the methodology of Dong et al [30] with modifications. The sORF2 protein was separated by SDS-PAGE and electroblotted to transfer it the surface of a polyvinylidene difluoride (PVDF) membrane. The primary antibodies were pig antisera and the secondary antibody was HRP-goat anti-swine IgG (BoAoSeng Company, Beijing, China). These pig antiseras were also tested using a commercial ELISA kit widely used to investigate seroprevalence of HEV infection in humans (Wantai Biological Pharmacy Company, Beijing, China) with replacement of HRP-goat anti-human IgG in the kit with HRP-goat anti-swine IgG. The coincidence rates of bELISA with iELISA and with Western blot and the commercial ELISA kit were calculated using Microsoft Excel’s CORREL function.

### Statistical analysis

Student's t-test and Kappa index values were calculated to estimate the differences in antigen binding blocking exhibited by the three HRP-mAbs, as well as the coincidence between bELISA and iELISA, between bELISA and western blot, and between bELISA with the commercial ELISA kit. These calculations were performed using SPSS software (Version 20, www.spss.com.cn).

## Result

### SDS-PAGE analysis of the purified antigen protein and mAbs

**Fig 1:**
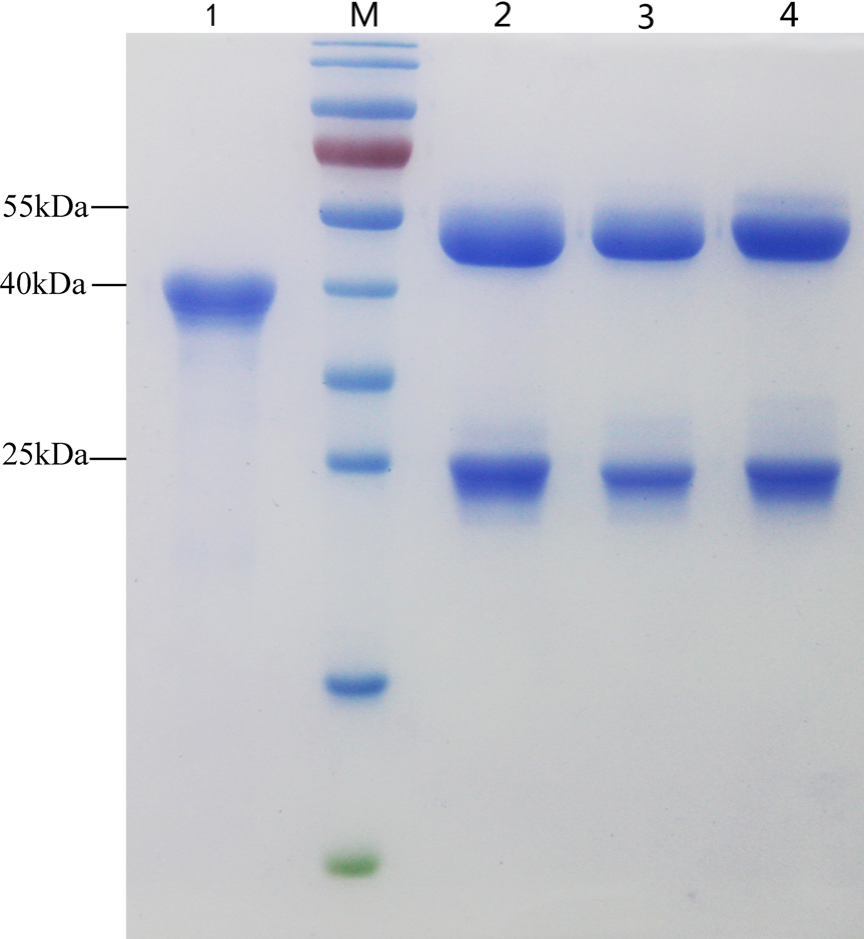
SDS-PAGE gel electrophoresis analysis of the recombinant sORF2-C fusion protein and three mAbs. The His-tagged sORF2-C fusion protein containing the C-terminal 268 amino acids of CHN-SD-sHEV was expressed in a bacterial system and purified using Ni-NTA resin. Three mAbs were produced using the sORF2-C protein as the immunizing antigen by traditional hybridoma technology. mAbs and sORF2-C were analyzed using SDS-PAGE. M: protein marker; lanes 1–4: purified sORF2-C protein, 1E4, 2C7 and 2G9. The loading quantity of each lane was 7.5 μg. The relative molecular masses of heavy and light chains were 50 and 25 kDa, respectively, and the sORF2-C protein was 40 kDa.

To produce the coating antigen for use in the bELISA, the recombinant sORF2-C protein was successfully expressed in a bacterial system and purified using a Ni-NTA resin column as an approximately 40 kDa His-tagged fusion protein (Fig 1, Lane 1). After purification, the concentration of the protein was determined using a Bradford Protein Assay Kit to be 7.0 μg/μL. Three mAbs, 1E4, 2C7 and 2G9, were produced using traditional hybridoma technology and purified using a goat-anti-mouse IgG affinity column. The concentrations of the three purified mAbs, 1E4, 2C7 and 2G9, were determined to be 1.86 μg/μL, 1.39 μg/μL, and 1.89 μg/μL, respectively, using a spectrophotometer. SDS-PAGE analysis result showed that the three mAbs had been purified successfully, with only heavy and light chains visualized in the gels using Coomassie blue staining (Fig 1, Lanes 2, 3 and 4).

### Titrations of three HRP-mAbs reacted with the sORF2-C protein

**Fig 2:**
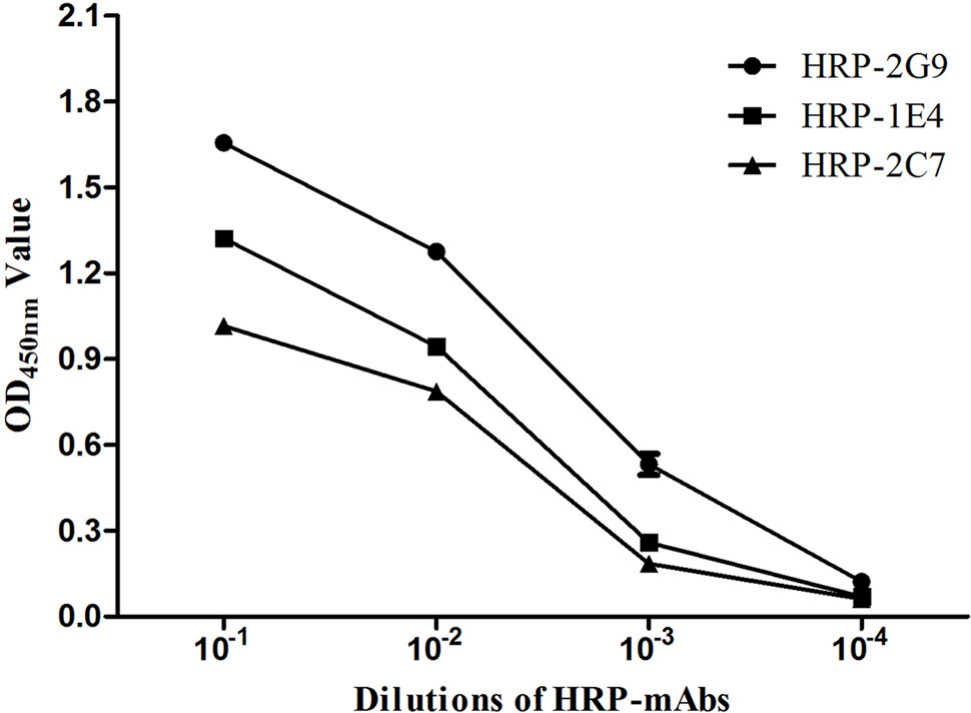
Analysis of the affinities of three HRP labeled mAbs (HRP-1E4, HRP-2C7, and HRP-2G9) for sORF2-C using direct ELISA. The three HRP-mAbs (1 mg/ml) in a dilution range of 10^−1^ to 10^−4^ were tested for reaction with the sORF2-C protein in the direct ELISA.

To titrate the three HRP-mAbs, ten serial dilutions of three HRP-mAbs (10^−1^ to 10^−4^) were assayed for binding to purified sORF2-C protein by direct ELISA. HRP-mAbs titers were defined as the highest serum dilutions that gave an OD_450nm_ value of 0.3 and the titers for HRP-1E4, HRP-2C7, and HRP-2G9 were 10^−2^, 10^−2,^ and 10^−3^, respectively (Fig 2).

### Relationships of epitopes recognized by three mAbs

The spatial relationships of epitopes recognized by the three mAbs were determined using a competitive ELISA. The results (Table 1) showed that the binding of HRP-1E4 to sORF2-C was inhibited by homologous 1E4, with a 68.92% inhibition value, while binding was not inhibited by 2C7 and 2G9, as evidenced by low respective PI values of 13.98% and 9.97%. The binding of HRP-2C7 to sORF2-C was inhibited by homologous 2C7, as evidenced by a PI value of 83.36%, but not by 2G9, as evidenced by a low PI of 16.59%. Results shown in Table 1 for all three mAbs indicate that mAbs 1E4, 2C7, and 2G9 recognize different epitopes.

**Table 1:** Inhibition of binding of HRP labeled mAbs to sORF2-C protein.

HRP labeled mAbs		PI value (%)*	
	1E4 (1:100)	2C7 (1:100)	2G9 (1:100)
HRP-1E4	68.92	13.06	18.94
HRP-2C7	13.98	83.36	16.59
HRP-2G9	9.97	15.05	70.21

* The PI values are calculated using the formula: 100 [1-(OD_450_ of HRP-mAb and mAb)/ (OD_450_ of HRP-mAb)].

### Selection of HRP-1E4 for developing the blocking ELISA

To select the optimal HRP-mAb from the three candidate HRP-mAbs for developing a bELISA, the 45 serum samples from the different dpi (0 to 56 dpi) of the 5 pigs challenged with CHN-SD-sHEV (genotype 4) were used to block the binding of the three HRP-mAbs to the sORF2-C protein. The results showed that, compared with HRP-2C7 and HRP-2G9, the PI ratios of HRP-1E4 (calculated from the ratio of PI for the 7 to 56 dpi serum samples to the PI of the 0 dpi sample) were the highest (both P values were <0.05) (Table 2). A range of PI ratio for the 5 individual pigs was shown in S1 Table. Therefore, HRP-1E4 was selected as the blocking mAb for the bELISA.

**Table 2:** Comparisons of the serial serum samples collected at different dpi of the five challenged pigs with CHN-SD-sHEV to block binding of the three HRP-mAbs to the sORF2-C protein.

HRP labeled mAb	Mean ratios of different dpi serum samples*								P value**
	7	14	21	28	35	42	49	56	
HRP-1E4	32.36	80.42	96.18	118.99	211.57	339.91	402.51	397.49	
HRP-2C7	6.95	10.11	20.47	63.89	106.32	154.53	174.16	194.42	0.003
HRP-2G9	1.78	4.63	8.11	35.07	44.56	56.41	58.44	57.59	0.006

* The ratios were the percent inhibitions (PI) of serial serum samples (7 to 56 dpi) / the PI of pre-challenged (0 dpi). The number is the mean of the results from five pigs.

** The testing results of the HRP-1E4 as the blocked antibody compared with the HRP-2C7 and HRP-2G9 were statistically analyzed.

### Development of the blocking ELISA with HRP-1E4

Using a checkerboard titration test, the optimal concentration of coated antigen and dilution of HRP-1E4 were determined to be 8 μg/ml and 1:10^3^, respectively, when the OD_450nm_ value was about 1.0 for each, as determined by direct ELISA (Table 3). In addition, the optimal dilution for the test serum was identified as 1:20 based on the optimal dilution of positive and negative serum samples producing the highest PI (Table 4).

**Table 3:** Optimized coating antigen concentration and HRP-1E4 dilution were determined by a checkerboard titration test with a direct ELISA.

Dilutions for HRP-1E4	OD_450nm_ value under different concentration of coating antigen (μg/ml)		
	2.0	4.0	8.0
1:100	1.416	1.552	2.068
1:1000	0.319	0.726	1.033
1:10000	0.105	0.291	0.478

**Table 4:** The optimized serum dilution was determined by bELISA with the positive and negative serum samples to block the HRP-1E4 reaction with the sORF2-C.

Serum dilution	OD_450nm_ value		PI value (%)*
	Positive serum	Negative serum	
1:10	0.46	1.03	55.34
1:20	0.41	0.99	58.59
1:40	0.64	0.97	34.02

* The PI ratios are calculated using the formula: PI (%) = [1 − (OD_450nm_ of positive serum /OD_450nm_ value of negative serum)] × 100%.

The cut-off value of the bELISA was determined by testing 230 negative pig serum samples by bELISA. The average PI (X) of the 230 negative samples was 6.4%, with a SD of 3.5%. The cut-off value of the bELISA was calculated to be 16.9% (6.4% + 3SD). Therefore, test serum samples with PI ≥ 16.9% and PI < 16.9% are designated positive and negative, respectively, using the bELISA.

### Sensitivity, specificity and reproducibility of bELISA

**Fig 3:**
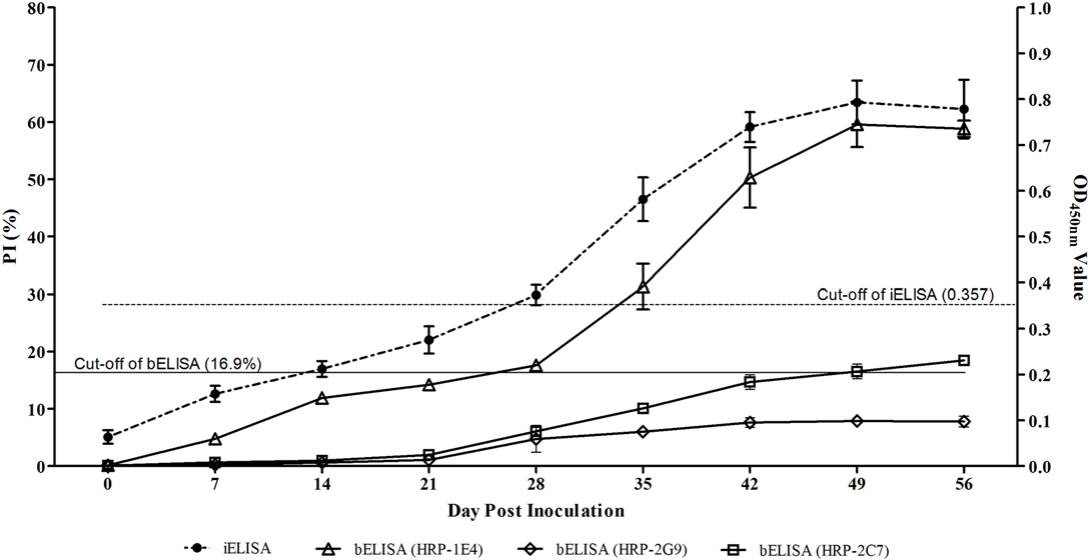
Comparisons of the detection results of anti-HEV antibodies in the serial serum samples from the five challenged pigs for iELISA and three bELISAs using HRP-1E4, HRP-2G9 and HRP-2C7 separately as blocking antibodies. The serum samples were collected at 0, 7, 14, 21, 28, 35, 42, 49 and 56 dpi from five pigs challenged with CHN-SD-sHEV. The presence of anti-HEV antibodies in the bELISA and iELISA were recorded as PI and OD_450nm_ values, respectively. The solid and dotted lines represent the cut-off values of the bELISA and iELISA, respectively. The pigs seroconverted at 28 dpi based on the cut-off values of the bELISA and iELISA.

**Fig 4:**
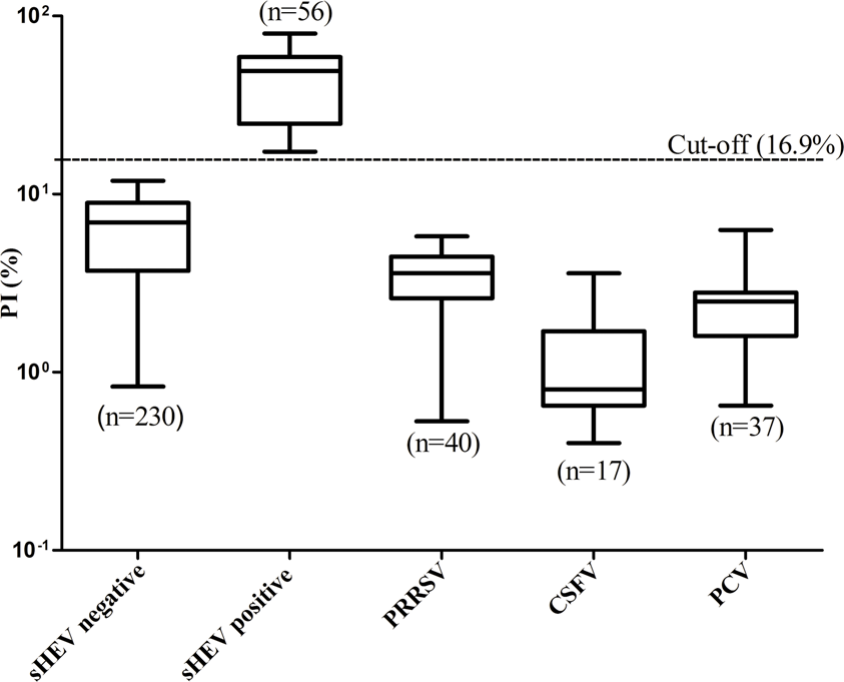
The cut-off value and specificity of the bELISA were determined using 230 HEV Ab-negative and 56 HEV Ab-positive pig serum samples. These samples were collected from pigs for which the presence of HEV antibodies was confirmed using a commercial ELISA kit for detecting anti-HEV antibodies in human sera. The cut-off value was set using the mean PI of 230 negative serum samples plus 3 SD (16.9%). The dotted line represents the cutoff value of the bELISA. The 40, 17, and 37 serum samples from PRRSV, CSFV and PCV positive pigs, respectively were collected for evaluating the specificity of the bELISA. The results for each group are shown as the range (whiskers), interquartile range (boxes), and median (line in the boxes). Abbreviations: sHEV, swine hepatitis E virus; PRRSV, porcine reproductive and respiratory syndrome virus; CSFV, classical swine fever virus; PCV, porcine circovirus.

To determine the sensitivity of the bELISA, the 45 serum samples from the pigs pre- and post-challenged with CHN-SD-sHEV, as well as 56 positive clinical serum samples, were tested. The 45 serum samples were first tested for antibodies against HEV by iELISA. The results showed that the pigs seroconverted from 28 dpi, based on the cut-off value of the iELISA (Fig 3). When these serum samples were tested using the bELISA developed here, seropositivity was also first observed at 28 dpi (Fig 3). For the 56 positive clinical pig serum samples which were confirmed using the commercial ELISA kit, seropositivity was confirmed for all based on the PI values detected by the bELISA (Fig 4). Therefore, based on the results of the serial serum samples from challenged pigs and clinical positive pig serum samples, bELISA exhibited sensitivity comparable to both iELISA and the commercial ELISA kit.

To test specificity using cross-blocking in the bELISA, antisera against three other major swine viruses, including PRRSV, CSFV and PCV, were also tested using the bELISA. The serum samples collected at 56 dpi from pigs challenged with CHN-SD-sHEV was used as a positive control. The results showed that the PI value of positive serum samples reached a maximum value of 67.7%, while the PI values of antisera against PRRSV, CSFV, and PCV were 3.59% ±4.25%, 1.25% ±5.01%, and 2.67% ±4.71%, respectively (Fig 4). The results showed the bELISA was specific for detection of HEV-specific antibody in domestic pigs.

The intra-assay and inter-assay variabilities were evaluated by comparing the PI of six positive serum samples which were tested repeatedly between bELISA plates and within a single plate. By testing the 6 serum samples in triplicate, the intra-assay coefficients of variation (CV) of the PI were observed to range from 0.84% to 2.71%, with a median value of 1.13% (Table 5, S2 Table). When the 6 samples were tested in three plates at different times, the inter-assay CV of the PI was observed to range from 2.12% to 5.23%, with a median value of 3.05% (Table 5, S2 Table). These data indicate that the bELISA method described in this study exhibits good reproducibility.

**Table 5:** The reproducibility of bELISA determined by CV % value of intra and inter assay.

		CV % value range of 6 samples	Median value
Intra assay precision (CV %)*	0.84–2.71		1.13
Inter assay precision (CV %) **	2.12–5.23		3.05

* Determined from triplicates readings (well-to-well) of 6 challenged serum samples in the same runs.

** Determined from triplicates readings (plate-to-plate) at different time.

### Agreements of bELISA with iELISA results with western blot and commercial ELISA kit results

When the 2,542 pig serum samples collected from different herds in Shaanxi Province were tested separately using bELISA and iELISA, the percentage of positive samples for bELISA was 8.1% (206/2,542) (Table 6) and for iELISA was 14.87% (378/2,542). The results of the bELISA and iELISA coincided in 2,370 of the 2,542 serum samples and the compliance rates were 93.23% (Table 6). From 2,336 bELISA antibody-negative serum samples, 172 samples were positive by iELISA (Table 6). Subsequently, 33 of the 206 samples testing positive for both bELISA and iELISA, as well as 117 of the inconsistent 172 samples that were seropositive by iELISA but negative by bELISA, were tested again using western blot and a commercial human anti-HEV ELISA kit. The results showed that the compliance rates of bELISA vs. western blot and bELISA vs. commercial ELISA kit were 92% and 95%, respectively (Table 6). For the 117 inconsistent serum samples, 12 samples were positive by western blot and 7 samples were positive by the commercial ELISA kit (Table 6). The 33 positive serum samples in both bELISA and iELISA were also positive by both western blot and the commercial ELISA kit (Table 6). In addition, statistical analysis showed the bELISA had a high level of consistency with iELISA (Kappa = 0.67), with western blot (Kappa = 0.79), and with the commercial ELISA kit (Kappa = 0.87) (Table 6). There were no significant differences between bELISA and iELISA, between bELISA and western blot, and between bELISA and the commercial ELISA kit (all Kappa values were >0.4).

**Table 6:** Comparisons of bELISA with iELISA, bELISA with western blot and bELISA with commercial ELISA kit by collected pig serum samples.

bELISA		Compared methods		Agreement (%)*	Kappa value**
		iELISA		93.23	0.677
		+	-		
+	206 (A)	206 (B)	0		
-	2336 (C)	172	2164 (D)		
		western blot		92	0.794
		+	-		
+	33 (A)	33 (B)	0		
-	117 (C)	12	105 (D)		
		Commercial ELISA kit		95	0.874
		+	-		
+	33 (A)	33 (B)	0		
-	117 (C)	7	110 (D)		

* Agreement (%) = (B+D) / (A+C) × 100

** Kappa values were calculated to estimate the agreements of the bELISA with iELISA, with western blot and with the commercial ELISA kit. The kappa value > 0.4 was regarded as significant difference.

## Discussion

HEV is a zoonotic virus with domestic and wild pigs considered to be a main reservoir for the virus [2]. Therefore, measuring the level of HEV infection in pig herds is an important step to prevent HEV infection in humans. At present, RT-PCR to detect HEV RNA and ELISA to detect anti-HEV antibodies are common methods for diagnosis of HEV infection and the status of the infection in pigs [32–35]. However, RT-PCR is technically complex, cumbersome, not cost-effective, and is sensitive to contamination. Therefore, the various in-house iELISAs developed by several researchers using coated antigens from swine HEV genotypes 3 or 4 and commercially available ELISA kits for detecting human anti-HEV antibodies have widely been used to detect pig anti-HEV antibodies [25, 26]. Nevertheless, the iELISA often produces non-specific background signals and has low coincidence and poor specificity due to use of different coated antigens from human HEV.

In the present study, a bELISA for detecting anti-HEV antibodies in pigs was successfully developed. The assay used the truncated ORF2 protein from CHN-SD-sHEV as coating antigen and HRP-1E4, a labeled mAb whose binding to the coating antigen was inhibited by anti-HEV antibodies in seropositive samples. The bELISA had a higher specificity than iELISA and the compliance rates of bELISA with iELISA, with western blot, and with a commercial human anti-HEV detecting ELISA kit were in high agreement. These results suggest that the bELISA developed herein can be used as a method to detect anti-HIV antibodies and investigate the status of HEV infection in the porcine herds. To our knowledge, the iELISAs have the limitations of the requirement of highly purified coating antigen and non-specific background signals. In contrast, the bELISA can overcome these limitations. This is true as the results in Table 6 showed that the bELISA had a higher specificity than iELISA.

In China, many serological investigations of the status of HEV infection in domestic pigs have been performed [36–39]. For example, previous studies documented that the prevalence of anti-HEV lgG antibodies was 66.4% in Shandong [28], 68.3% in Hunan [40], 63.9% in Shanghai and Jiangsu [41], and an average prevalence of 78.8% in Beijing, Henan, Zhejiang, Guangdong and Hubei [42]. These previous investigations suggested that HEV infection is endemic in domestic pig populations in some regions of China. However, in the present study, the positive rate was only 8.1% (based on the detection results of bELISA) or 14.57% (based on the iELISA) in the domestic pigs of Shaanxi Province, which is significantly lower than the positive rate detected in the eastern and southern regions of China. The reasons may lie in the fact that the pork industry in the Shaanxi Province began more recently, is still of small scale, and incorporates the highest biosafety practices. Therefore, it is advantageous to the pork industry to encourage higher biosafety efforts to prevent HEV virus infection.

Conventionally, when a new assay is developed it is a common practice to compare the new assay with current “gold standard” assays to assess the value of the new assay. However, there is no highly effective in vitro cell culture protocol for HEV propagation and no “gold standard” assay is available for diagnosing HEV infection in domestic pigs. Prior to this study, detection of anti-HEV antibodies in pigs universally relied on either a commercial ELISA kit optimized for human serum sample testing or in-house iELISAs using the ORF2 and ORF3 proteins from genotypes 3 or 4 HEV as coating antigens.

In this study, differences and agreements were analyzed between the bELISA developed here and the widely used iELISA, western blot, and a commercial ELISA kit used in China for testing for human anti-HEV antibodies. The data showed high coincidence between bELISA and iELISA (Kappa = 0.67), between bELISA and western blot (Kappa = 0.79), and between bELISA and the commercial ELISA kit (Kappa = 0.87). These results suggest that the bELISA is more reliable than the other tests and can replace the iELISA, western blot, and the commercial ELISA kit to detect anti-HEV antibodies in domestic pigs.

In summary, a bELISA was developed for detecting anti-HEV antibodies in domestic pigs using the truncated ORF2 protein from CHN-SD-sHEV (genotype 4) as coating antigen and HRP-1E4 as labeled antibody whose binding to coating antigen is blocked by unlabeled antibodies in the seropositive samples to be tested. The assay shared the same level of sensitivity with the iELISA and the commercial ELISA kit, as well as higher specificity for detection of porcine anti-HEV antibody levels as compared to iELISA. In addition, the results of the bELISA exhibited high reproducibility and good agreement with iELISA, western blot, and the commercial human serum ELISA kit. Therefore, we believe that the bELISA should serve as an ideal method for large-scale serological studies of HEV infection in domestic pigs.

## Supporting Information

S1 Table A range of PI ratio for 5 individual pigs. (DOCX)

S2 Table The intra-assay and inter-assay variability. (DOCX)
