# Supplementary material for: A Novel Blocking ELISA for Detection of Antibodies against Hepatitis E Virus in Domestic Pigs
Source: PLoS One. 2016 Mar 29;11(3):e0152639. doi: 10.1371/journal.pone.0152639 (PMC4811412; doi:10.1371/journal.pone.0152639)
Supplement: S1 Table — (DOCX) [file pone.0152639.s001.docx]

**Supporting Information**

**S1 Table. A range of PI ratio for 5 individual pigs.**

| **mAb** | **The range of PI ratio(%) for five individual pigs from 0 to 56(dpi)** | | | | | | | | |
| --- | --- | --- | --- | --- | --- | --- | --- | --- | --- |
|  | 0 | 7 | 14 | 21 | 28 | 35 | 42 | 49 | 56 |
| HRP-1E4 | 0.16±0.05 | 4.87±0.75 | 12.30±2.09 | 14.17±1.95 | 17.48±1.86 | 26.73±9.75 | 44.37±14.05 | 59.28±10.60 | 58.58±3.74 |
| HRP-2C7 | 0.10±0.01 | 0.66±0.23 | 0.96±0.18 | 1.95±0.71 | 6.07±0.86 | 10.10±0.24 | 14.68±1.26 | 16.55±1.29 | 18.47±0.60 |
| HRP-2G9 | 0.14±0.01 | 0.24±0.10 | 0.63±0.17 | 1.10±0.16 | 4.74±2.35 | 6.02±0.35 | 7.62±1.02 | 7.89±0.46 | 7.78±0.94 |
