# Supplementary material for: A Novel Blocking ELISA for Detection of Antibodies against Hepatitis E Virus in Domestic Pigs
Source: PLoS One. 2016 Mar 29;11(3):e0152639. doi: 10.1371/journal.pone.0152639 (PMC4811412; doi:10.1371/journal.pone.0152639)
Supplement: S2 Table — (DOCX) [file pone.0152639.s002.docx]

**Supporting Information**

**S2 Table.** **The intra-assay and inter-assay variability.**

|  | **The range of PI ratio (%) of six positive serum samples** | | | | | |
| --- | --- | --- | --- | --- | --- | --- |
|  | 1 | 2 | 3 | 4 | 5 | 6 |
| Intra-assay | 17.23±0.15 | 30.11±0.45 | 38.24±0.34 | 42.30±0.88 | 51.20±0.92 | 58.77±0.65 |
| Inter-assay | 17.75±0.47 | 29.84±0.72 | 38.82±1.80 | 43.40±1.02 | 51.93±1.67 | 58.97±1.33 |
